# Antibacterial electrospun chitosan‐based nanofibers: A bacterial membrane perforator

**DOI:** 10.1002/fsn3.468

**Published:** 2017-04-10

**Authors:** Mounia Arkoun, France Daigle, Marie‐Claude Heuzey, Abdellah Ajji

**Affiliations:** ^1^ CREPEC Department of Chemical Engineering École Polytechnique de Montréal Montréal QC Canada; ^2^ Department of microbiology infectiology and immunology Pavillon Roger‐Gaudry Université de Montréal Montréal QC Canada

**Keywords:** antibacterial, electrospun chitosan‐based nanofibers, membrane perforation, membrane permeability

## Abstract

This study investigates the antibacterial action of chitosan‐based nanofibers (CNFs) obtained by the electrospinning process on the permeability of bacterial membranes. The bactericidal efficiency of CNFs was first determined against Gram‐negative *Escherichia coli* and *Salmonella* Typhimurium, and Gram‐positive *Staphylococcus aureus* and *Listeria innocua* bacteria as a baseline. The results strongly suggest that CNFs interact with the negatively charged bacterial cell wall causing membrane rupture and inducing leakage of intracellular components among which are proteins and DNA. Results clearly indicate that the release of such components after contact with CNFs is an indication of membrane permeabilization and perforation, as pore formation was observed in transmission electron microscopy (TEM). This work suggests a plausible antibacterial mechanism of action of CNFs and also provides clear evidence in favor of chitosan as a bacterial membrane disruptor and perforator. As a result, CNFs can find promising applications as bioactive food packaging materials capable to extend shelf life of food products while inhibiting the spread of alteration flora and foodborne pathogens.

## 1 INTRODUCTION

Electrospinning of chitosan, with the aim of producing nanofibers with diameters ranging from few tens of nanometers to micrometers, has been the subject of several recent studies (Desai, Kit, Li, & Zivanovic, 2008; Doğan, Özyıldız, Başal, & Uzel, 2013; Elsabee, Naguib, & Morsi, 2012; Geng, Kwon, & Jang, 2005; Homayoni, Ravandi, & Valizadeh, 2009; Kriegel, Kit, McClements, & Weiss, 2009; Pakravan, Heuzey, & Ajji, 2011; Rieger, Birch, & Schiffman, 2016; Ziani et al., 2011). The resulting chitosan nanofiber (CNF) mats exhibit a remarkably high porosity (in the range of 80%–90%) and surface area per unit mass (between 10 and 500 m^2^/g) and display good biocompatibility and biofunctionality (Ardila et al., 2016; Greiner & Wendorff, 2007). Therefore, CNFs may have promising applications in biomedical (cell culture, wound healing, tissue engineering) (Ignatova, Manolova, Markova, & Rashkov, 2009), pharmaceutics (controlled drug release, gene therapy) (Jayakumar, Prabaharan, Nair, & Tamura, 2010), water filtration (chelation of metal ions) (Haider & Park, 2009), and food packaging (Martínez‐Camacho et al., 2011), among others. However, achieving high yield and quality fiber formation from neat chitosan solutions is a challenging task. This is mainly due to the very rigid structure of chitosan chains, which does not promote entanglements that are required for the formation of the Taylor cone, which in turn generates nanofibers. For example, some authors reported the preparation of neat CNFs using trifluoroacetic acid (TFA) as a solvent or its mixtures with dichloromethane (DCM) (Gu et al., 2013; Lee et al., 2014). However, TFA is highly cytotoxic, corrosive, and environmentally harmful, making the use of such materials incompatible with applications as delicate as food packaging. Moreover, electrospinning is a multifactorial process that involves several parameters among which processing conditions such as flow rate, electric field, collecting distance, temperature and humidity, as well as intrinsic solution parameters including conductivity, surface tension, and viscoelasticity. Thus, in order to improve the electrospinnability of chitosan, a cospinning agent at moderate content is often needed and used as a carrier polymer to trigger fiber formation (Moayeri & Ajji, 2015; Rieger et al., 2016).

Studies have demonstrated that chitosan, in the form of solution and films, exhibits efficient antimicrobial activity (Muzzarelli et al., 1988; Papineau, Hoover, Knorr, & Farkas, 1991; Shahidi, Arachchi, & Jeon, 1999; Sudarshan, Hoover, & Knorr, 1992; Young, Köhle, & Kauss, 1982). However, few have examined the antibacterial properties of CNFs. In a review article, Martínez‐Camacho et al. (2011) point out that most reports on the antimicrobial activity of CNFs have used chitosan solutions instead. In most cases, the proposed mechanism for CNFs was indirectly related to the presence and release of protonated amino groups from CNFs mats, which were no longer nanofibers. The authors highlighted that further investigation would be useful in order to determine whether CNFs follow the same presumed mechanism, since it might be affected by the structural conformation these nanomaterials can adopt (Kong et al., 2008). The mechanism of action by which chitosan, in solution state, is able to inhibit or kill bacteria is a complex phenomenon that has not been fully explained either (Hammer et al., 2010; Kong, Chen, Xing, & Park, 2010; Raafat, Von Bargen, Haas, & Sahl, 2008). Moreover, no information is available regarding the mechanism underlying the antimicrobial activity of CNFs. To our knowledge, no study has reported the effect of CNFs on bacterial cell membrane integrity, nor their mode of action. A cytological study of the effect of CNFs on the bacterial membrane permeability is necessary to understand their exact mechanism of action and to avoid the outbreak of potential resistance phenomena. In this study, we investigate the antibacterial mechanism of action of CNFs against four common alteration flora and foodborne pathogens, most frequently incriminated in food spoilage and food poisoning, respectively. All tests were performed under standardized and controlled experimental conditions to facilitate reproducibility and allow comparative studies. A plausible mode of action in which CNFs act as membrane permeability disruptor and even perforator is postulated. In this context, CNFs represent ideal biomaterials that can be used as suitable bactericidal barriers to prevent bacterial infections in several areas, including food packaging and biomedical applications. As part of active food packaging, CNFs can be applied to extend the shelf life of food products and prevent spoilage and foodborne diseases caused by *Escherichia coli, Listeria*,* Staphylococcus,* and *Salmonella*.

## 2 MATERIALS AND METHODS

### 2.1 Chemicals and polymers

Water‐soluble chitosan (CS), a Venzym^™^ grade obtained *via* enzymatic treatment of chitin derived from shrimp shells was generously donated by Ovensa (Ontario, Canada). The water‐solubility of this CS grade is due to the presence of a low amount of residual acetic acid (AcOH), as confirmed by the supplier. The corresponding degree of deacetylation (DDA) and number average molecular weight (M_n_) are 95% and 50 kDa, respectively, with a narrow molecular weight distribution. Poly(ethylene oxide) (PEO), a cospinning agent for chitosan, with a molecular weight of 600 kDa, and acetic acid (AcOH, glacial, 99.7%) were purchased from Fisher Scientific (Saint‐Laurent, QC, Canada). All materials were of analytical grade and used as received.

### 2.2 Microorganisms, culture media and conditions

#### 2.2.1 Bacterial strains

*Escherichia coli* (DH5α), *Salmonella* Typhimurium (SL1344), *Staphylococcus aureus* (54‐73), and *Listeria innocua* (ISPQ3284) were supplied by the Laboratory of Microbiology, Université de Montréal (Québec, Canada). Cultures were maintained at 4°C prior to use, then transferred into a culture medium and finally incubated at 37°C for 24 hr in an orbital shaker (New Brunswick) to achieve an initial concentration of 10^9^ colony forming unit per milliliter (CFU/ml).

#### 2.2.2 Culture media

Luria–Bertani (LB) broth and brain heart infusion (BHI) were used as growth media to start the bacterial cultures. To reach the required final concentration, cultures were diluted using phosphate buffer saline (PBS, pH 5.8, adjusted with 1 mol/L HCl). LB agar and BHI supplemented with agar (15 g/L) were used as solid media for counting the surviving bacteria.

### 2.3 Preparation of chitosan and PEO stock solutions

Chitosan (CS) and PEO stock solutions (7% w/v and 3% w/v, respectively) were individually prepared by dissolving polymer powders in 50% (v/v) AcOH under overnight magnetic stirring. The CS/PEO blends were obtained by magnetic stirring of the two polymer solutions in a proportion of 80/20 (w/w) ratio for 4 hr agitation. The advantage of using aqueous acetic acid solutions is their nontoxic and ecofriendly character.

### 2.4 Preparation of chitosan‐based nanofibers *via* electrospinning

**Figure 1:**
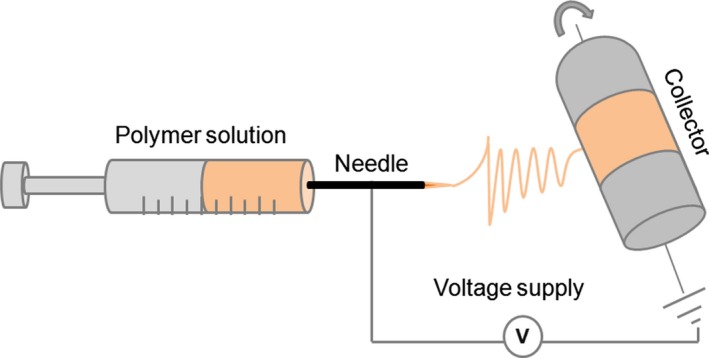
Schematic representation of the homemade electrospinning set up

CS/PEO nanofibers were prepared according to Pakravan et al. (2011) using the electrospinning process. Electrospinning of the blend solution was performed using a horizontal homemade setup containing (1) a high voltage power supply (Gamma High Voltage Research, FL, USA), (2) a programmable pump (Harvard Apparatus, PHD 2000) to deliver the polymer solution at the required flow rate, and (3) a metallic rotating drum wrapped with an aluminum foil to collect the nanofibers. A schematic representation of the set up is shown in Figure 1. The electrospinning blend solution was poured into a 10 ml syringe with Luer–Lock connection to an 18‐gauge blunt tip needle (Cadence Science, USA). The syringe was mounted on the pump with a grip and grounded by use of an alligator clip. The optimal process parameters were flow rate of 0.5 ml/hr, voltage of 20 kV, and needle tip‐to‐collector distance of 15 cm. All experiments were conducted at room temperature (22 ± 1°C), relative humidity of 20%, and under atmospheric pressure. The collected nanofibers were dried overnight under a hood to ensure complete evaporation of the solvent.

### 2.5 Scanning electron microscopy (SEM)

The morphology of the electrospun chitosan‐based nanofibers (CNFs) was examined according to a slight modified method of Moayeri and Ajji (2015), using a field emission scanning electron microscope (FESEM JEOL JSM‐7600TFE), operated at 1.5 kV. Samples were observed as collected on an aluminum foil after 2 hr electrospinning. SEM results revealed that uniform and beadless fibers were obtained in the presence of the cospinning agent, PEO in this specific case. The average fiber diameter was evaluated using Image‐Pro Plus^®^ software. Approximately 600 nanofibers randomly chosen from three independent electrospun mats (200 fibers from each sample) were used for the quantification of fiber morphology parameters.

### 2.6 Antibacterial efficiency of CNFs

The antibacterial activity of electrospun CNFs was evaluated in vitro following the American standard test method (ASTM E2149−13a, 2013). Commonly found bacteria, *E*. *coli*,* S. aureus*,* L. innocua*, and *S. *Typhimurium, in food contamination and skin infections were selected for this purpose. Samples of 1 cm^2^ and 2.5 cm^2^ swatches of CNFs were prepared in aseptic conditions. Bacterial suspensions (10^6^ CFU/ml, 5 ml PBS, pH 5.8) were put in contact with CNFs. It is noteworthy that even though the CS grade used in this study was water‐soluble, the resulting nanofibers were visually insoluble in aqueous media post‐electrospinning due to solvent evaporation during processing. Negative controls of bacteria suspended in PBS without CNFs were also prepared. All tubes were placed at 37°C, optimal temperature for bacterial growth, for 4 hr incubation in an orbital shaker. Serial dilutions were performed and spread on agar plates incubated overnight at 37°C for further counting of survivors. All tests were conducted in triplicate. Finally, the antibacterial efficiency was expressed as a function of the reduction rate (*R*) of the total number of test bacteria. *R* was calculated according to Belalia, Grelier, Benaissa, and Coma (2008) using the following equation:

(1) $$R\left(\%\right)=\frac{A-B}{A}\times 100$$

where, *A* and *B* are the numbers of surviving bacteria in the controls and test samples, respectively.

### 2.7 Effect of chitosan‐based nanofibers on membrane permeability

#### 2.7.1 Sodium dodecyl sulfate‐polyacrylamide gel electrophoresis (SDS‐PAGE)

The release of intracellular proteins from CNF‐treated bacteria was investigated by SDS‐PAGE. In this section, *E. coli* (Gram‐negative) and *S. aureus* (Gram‐positive) were selected in order to appraise the effect of Gram‐type on the strains’ susceptibility/resistance to CNFs. Overnight cultures of *E. coli* and *S. aureus* were resuspended in PBS (~10^8^ CFU/ml) and incubated at 37°C in the presence of CNFs. After 0, 1, 2, 3, and 4 hr contact time, 5 ml aliquots were withdrawn and centrifuged at 3,000 *g*/10 min at 4°C. The supernatants were then mixed with trichloroacetic acid (TCA 10:1) and left for precipitation at 4°C overnight. After a series of wash, samples were resuspended in SDS‐loading buffer and subjected to SDS‐PAGE according to the method of Laemmli (1970). Positive controls (Ctrl^+^) of extracted proteins from *E. coli* and *S. aureus* were also prepared by chemical lysis of both bacteria using a lysis solution containing 50 μl of chloroform and 25 μl of SDS (0.5% v/v). For more sensitivity, revelation was performed using silver nitrate staining of proteins.

#### 2.7.2 Agarose gel electrophoresis of released DNA

Because of its importance in fundamental research, its use in the industrial field and its involvement in the agri‐food sector, the *E. coli* laboratory strain has been fully sequenced and its genome is currently 100% known. In the following section, *E. coli* (DH5α) bacterium was chosen to study the effect of CNFs on membrane permeability and subsequent DNA leakage.

The leakage of DNA from CNF‐treated *E. coli* was investigated by agarose gel electrophoresis as an indication of membrane damage. DNA was extracted from CNF‐treated *E. coli* cultures according to the protocol of Green and Sambrook (2012). Briefly, 5 ml aliquots were subjected to centrifugation (6,000 rpm, 10 min at 4°C), filtration (0.22 μm pore size) and overnight precipitation at −20°C in sodium acetate (NaAc 3 mol/L pH 5.2) and ethanol (EtOH 100%, −20°C, 2.5 × volume). Samples were centrifuged (9,000 *g*, 15 min, 4°C) and the resulting pellets were suspended in ethanol (70%, −20°C), centrifuged again, dried under the hood and resuspended in milliQ water. Positive controls of bacterial DNA extracted from *E. coli* after chemical and heat treatment (CtrlL^+^ and CtrlH^+^, respectively) were also prepared. An additional step of pH adjustment (pH 7.0) with 1 mol/L NaOH in order to deprotonate the CNFs and break up CS‐DNA interactions was necessary. A polymerase chain reaction (PCR) for the *rrnB* gene 16S RNA was performed in order to amplify the released DNA fragments from chitosan‐treated cultures. Finally, DNA extracted sequences were loaded on a 2% (w/v) agarose gel and migrated for 20 min at 90 V. DNA quantification was also performed using a NanoDrop spectrophotometer (ND‐1000, Thermo Scientific).

#### 2.7.3 β‐Galactosidase assay

In this section, *E. coli* DH5 hxt 55632–*Lac Z* ^*+*^, a strain that overexpresses the gene encoding the β‐galactosidase (β‐gal) activity (without addition of lactose to the medium) was selected to assess the effect of CNFs on membrane permeabilization. To this end, the release of intracellular β‐gal was evaluated by enzymatic titration according to Miller (1992). An overnight culture was diluted in LB and brought to an optical density (OD_600_) of 0.6, using a spectrophotometer (Spectrotonic 200; ThermoFischer). The suspension was then incubated at 37°C, in the presence (treated samples) and absence (negative control, Ctrl^−^) of CNFs at different contact times. A positive control (Ctrl^+^) of lysed cells was prepared by adding 50 μl of chloroform and 25 μl of SDS (0.1% v/v) to the culture. A volume (*v*) of 50 μl of each sample was diluted in 950 μl of neutral buffer (Z buffer, pH 7.0) over an ice bath. Samples were placed for 5 min at 28°C in a water bath before starting the reaction. To each sample, 200 μl of *o*‐nitrophenyl‐β‐galactoside (ONPG, 4 mg/ml) was added and the reaction was timed. When samples turned yellowish, the reaction was stopped by adding 500 μl of 1mol/L Na_2_CO_3_ and the time recorded (*t*). Tubes were then centrifuged 2 min at 13,000 *g* to remove cell residues and the optical density of the supernatant was measured at 420 nm and 550 nm (OD_420_ and OD_550_). Finally, the β‐galactosidase activity, expressed in β‐gal units or Miller units was calculated using the following equation:

(2) $$\beta -\text{galactosidase units}=\frac{1000\times ({\text{OD}}_{420}-1.75\times {\text{OD}}_{550})}{t\times v\times {\text{OD}}_{600}}$$

### 2.8 Transmission electron microscopy analysis of bacterial membrane integrity

Transmission electron microscopy (TEM) was performed to investigate the effect of CNFs on cell morphology and membrane integrity. Sample preparation was performed following the guidelines of Tao, Qian, and Xie (2011) and Xing et al. (2009a) with a slight modification. Overnight cultures (10^6^ CFU/ml) of the selected bacteria were exposed to CNFs for 10, 20, and 30 min. Cultures were then centrifuged (6,000 *g*/3 min) and the resulting pellets were resuspended in a 2% (v/v) glutaraldehyde solution contained in PBS (pH 7.4) for overnight fixation of the cells at 4°C. A quantity of 10 μl of each sample was deposited on Formvar carbon‐coated grids containing one drop of 1% Alcian Blue. Cells were then subjected to 5 min post‐fixation with 2% paraformaldehyde in PBS, and grids were stained using a drop of filtered 2% phosphotungstic acid (PTA, pH 7.0) for 30 s. A series of filtration and/or washing treatment was performed after each step to remove excess liquid, fixative, and staining. Untreated bacteria samples were also prepared by the same method. Finally, TEM observation was performed using a Philips CM100 transmission electron microscope (Philips Electron Optics, Eindhoven, The Netherlands) and digital micrographs were captured using an AMT XR80 CCD digital camera (Advanced Microscopy Techniques, Woburn, MA USA).

## 3 RESULTS AND DISCUSSION

### 3.1 Morphology of electrospun CNFs

**Figure 2:**
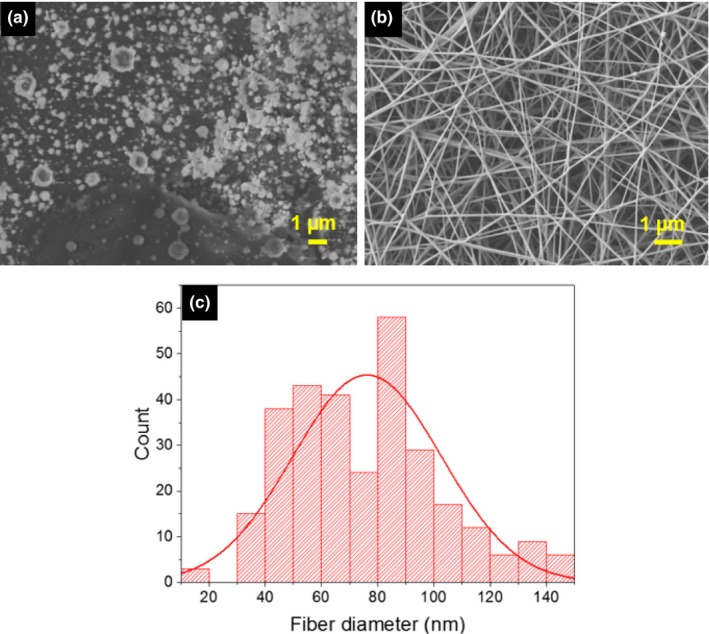
SEM micrographs of (a): electrosprayed 7% (w/v) CS in 50% (v/v) AcOH and (b): electrospun 7% (w/v) CS/PEO (80/20) in 50% (v/v) AcOH at 21°C, 7% relative humidity, (c): Fiber diameter distribution of b. Process parameters: tip‐to‐collector distance = 15 cm, flow rate = 0.5 ml/hr, voltage = 20 kV. Scale bars represent 1 μm diameter and magnification ×6 and ×10 for samples 2a and 2b, respectively

Figure 2 shows SEM images of electrospun CNFs from 7% (w/v) CS solution in 50% (v/v) AcOH, and 80/20 wt ratio CS/PEO blend in 50% (v/v) AcOH. As shown in Figure 2a, electrospinning neat CS was a difficult task and mostly gave rise to particles of nanometer and micrometer size (electrospraying). Hence, the addition of a cospinning agent, such as PEO, to facilitate the electrospinnability of CS was unavoidable. When added to CS solution in a moderate proportion (CS/PEO wt ratio: 80/20), PEO could act as a carrier and improve the viscoelastic properties of CS solution as well as chain entanglement and flexibility (Pakravan et al., 2011), two *sine qua non* conditions for fiber formation. Consequently, homogeneous and beadless chitosan‐based nanofibers with average fiber diameter of 78 nm ± 22 were successfully obtained.

### 3.2 Antibacterial efficiency of CNFs

**Figure 3:**
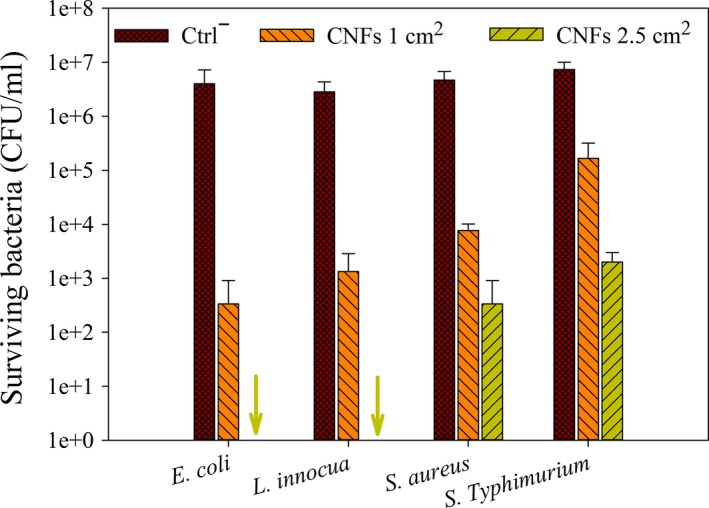
Antibacterial activity of CNFs against *E. coli*,* L. innocua*,* S. aureus*, and *S. *Typhimurium, as evaluated by the dynamic CFU method after 4 hr incubation at 37°C in PBS (pH 5.8). The arrows point at the complete inhibition of bacterial growth (*R* = 100%)

Table 1 and Figure 3 display, respectively, the bacterial reduction rate (*R*) and the in vitro antibacterial activity of CNFs, quantitatively assessed by the CFU method against *E. coli*,* S. *Typhimurium, *L. innocua*, and *S. aureus*. After 4 hr contact at 37°C in PBS (pH 5.8), CNFs (1 cm^2^) showed significant reduction rate (*R *>* *99%) of bacterial growth of *E. coli*,* L. innocua*, and *S. aureus* (Table 1), versus 96.91% for *S. *Typhimurium. When CS content was increased (2.5 cm^2^ instead of 1 cm^2^), CNFs were able to completely stop the growth of *E. coli* and *L. innocua*, (100% *R*, Table 1), as shown by the arrows (Figure 3). However, *S. aureus* and *S. *Typhimurium showed lower susceptibility to the action of CNFs. Nevertheless, a significant dose‐dependent decrease of bacterial population (5 logs and 4 logs, respectively) was still observed (Figure 3). Furthermore, in order to increase the anti‐salmonella or anti‐staphylococcal activity of CNFs, it is possible to combine chitosan with other antimicrobial agents such as ethylenediamine tetraacetic acid (EDTA, 0.2%) (Olaimat & Holley, 2015) and essential oils (Shahbazi & Shavisi, 2016), for a synergistic effect.

**Table 1:** Bacterial reduction rate (R) of CNFs against *E. coli*,* S. *Typhimurium, *L. innocua*, and *S. aureus*, as quantitatively assessed by the CFU method, after 4 hr incubation at 37°C in PBS (pH 5.8)

Reduction rate (%)				
Nanofiber webs	*E. coli*	*S. *Typhimurium	*S. aureus*	*L. innocua*
1 cm^2^ CNFs	99.93 ± 0.5	96.81 ± 2.3	99.14 ± 1.8	99.90 ± 0.02
2.5 cm^2^ CNFs	100 ± 0	98.97 ± 1,2	99.98 ± 0,5	100 ± 0

### 3.3 Proteins leakage

**Figure 4:**
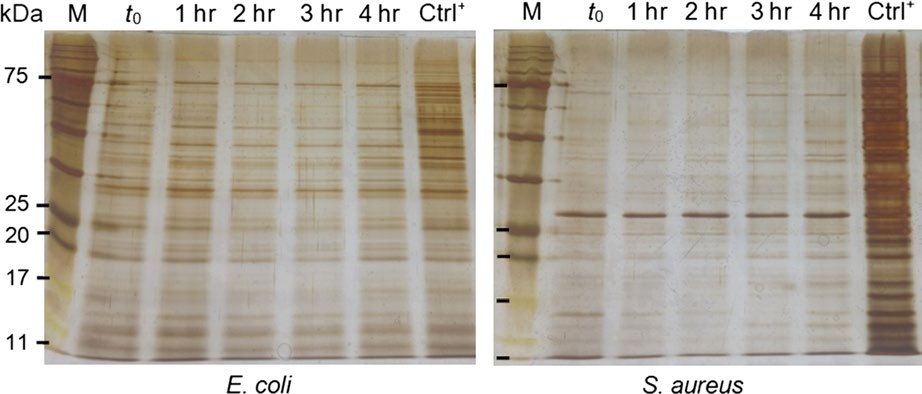
SDS‐PAGE patterns of released intracellular proteins from CNF‐treated *E. coli* and *S. aureus*, after 0, 1, 2, 3, and 4 hr contact time at 37°C in PBS. Ctrl^+^ refers to total proteins chemically extracted after treatment of cells with a lysis solution containing 50 μl chloroform and 25 μl SDS (0.5% v/v)

The release of intracellular proteins is an indication of membrane deterioration. Figure 4 shows SDS‐PAGE patterns of released cytoplasmic soluble proteins from chitosan‐treated *E. coli* and *S. aureus*. In the case of *E. coli*, the protein content in the cell‐free supernatant was similar to that of the positive control (Ctrl^+^) that refers to bacterial suspension after cell lysis treatment. This result indicates that the effect of CNFs was instantaneous (in the first hour of treatment) and almost fully completed since all intracellular proteins were released to the extracellular medium, as judged by the comparison between the CNF‐treated and the chemically lysed samples. For *S. aureus* bacterium, the effect was gradual. The electrophoresis pattern showed that the intensity of the bands increased with time, indicating that protein leakage was longer and progressive. When compared to the positive control (Ctrl^+^), the effect of CNFs was incomplete and several bands did not appear even after 4 hr exposure. This indicates that many proteins remained in the cytoplasm of living cells. These results suggest that chitosan plays an active role in membrane permeabilization. However, the observed antibacterial effect of CNFs on membrane damage, as reported by protein release was more pronounced in the case of *E. coli* than *S. aureus*, suggesting a higher susceptibility of *E. coli*, as reported in another study (Arkoun, Daigle, Heuzey, & Ajji, 2017).

### 3.4 DNA leakage

**Figure 5:**
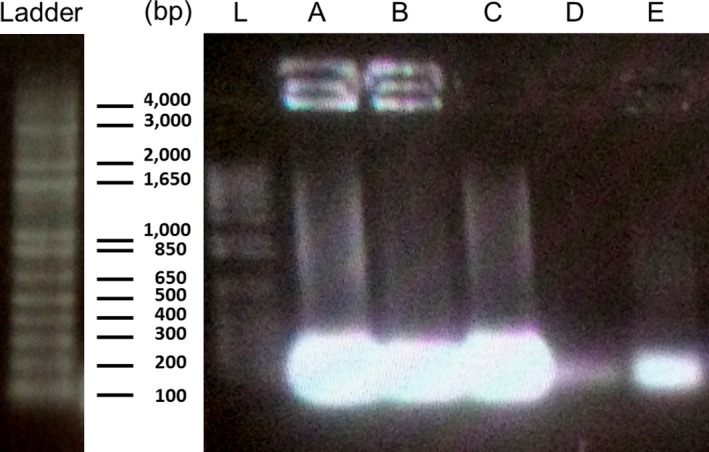
Agarose gel electrophoresis of released genomic DNA from CNF‐treated *E. coli* after A: 4 hr and B: 24 hr contact time. Samples C and E refer to CtrlL^+^ and CtrlH^+^ positive controls of *E. coli* DNA after chemical and heat treatment of bacterial cells, respectively. Sample D refers to negative control of genomic DNA extracted from untreated bacterial cells. L refers to ladder's fragments whose molecular weights are given in base pair (bp)

The release of bacterial genomic DNA in the supernatant was detected by PCR amplification of the *rrnB* gene (16S) for *E. coli* (Figure 5). The additional step of pH adjustment to neutrality mentioned in the methodology was necessary to hinder complexation of DNA with CNFs. Otherwise, no trace of the former could be detected. Detection of DNA in the extracellular medium (supernatant) was a consequence of the disruption of membrane permeability caused by CNFs (Figure 5A and B). In contrast, no DNA was detected in the extracellular medium of untreated sample (Ctrl^−^, Figure 5D), which was synonymous with membrane integrity. The observed brightness at the loading spots of the treated samples was probably due to a deposition of small cationic chains of CS itself, which did not migrate towards the cathode. This can be also attributed to a deceleration of the electrophoretic mobility of genomic DNA caused by the chelation effect of chitosan, as suggested by Xing, Chen, Liu, Cha, and Park (2009b). Negatively charged phosphate groups present in nucleic acids, such as DNA and RNA, might be an intracellular target for CS and contribute to its interaction with bacterial cells. This conjecture was verified when CS was deprotonated (at neutral pH) in order to prevent CS‐DNA complexation. As a consequence, genomic DNA was detected both qualitatively and quantitatively. These results point out that the leakage of bacterial DNA would not occur without membrane perforation and strongly suggest a membranolytic effect in CNFs’ mechanism of action. The concentrations of released DNA after exposure to CNFs, as measured using a NanoDrop spectrophotometer (ND‐1000, Thermo Scientific), after PCR were 18.2, 19.5, 20.9, 60.2, and 172.3 ng/μl, after 0, 1, 2, 3, and 4 hr exposure times, respectively. Quantification of released DNA from CNF‐treated *E. coli* clearly indicates that genomic DNA could be detected in the extracellular medium and its concentration was proportional to the contact time between *E. coli* and CNFs.

### 3.5 Release of intracellular β‐galactosidase enzyme

**Figure 6:**
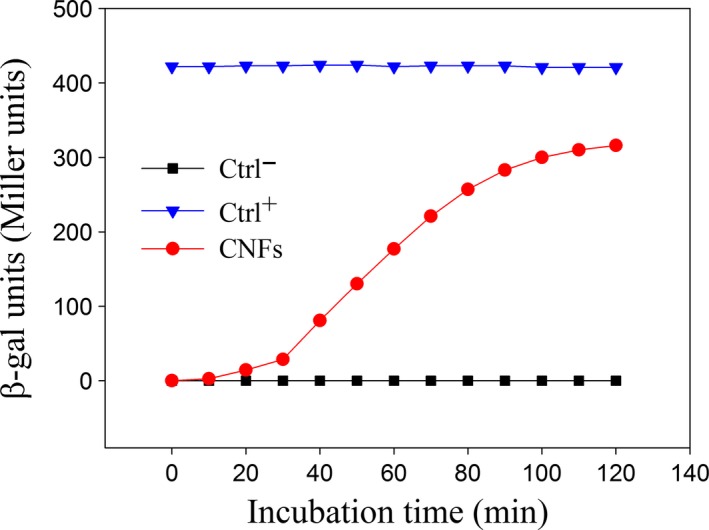
Release of cytoplasmic β‐galactosidase (β‐gal) enzyme from *E. coli* DH5 hxt 55632–*Lac Z* ^*+*^, after different exposure time to CNFs. Ctrl^−^ (negative control) refers to the level of released β‐gal in the absence of CNFs. Ctrl^+^ (positive control) refers to the level of β‐gal released by chemically lysed cells (prepared by adding 50 μl of chloroform and 25 μl of SDS to the culture)

The release of cytoplasmic β‐galactosidase (β‐gal) was also an evidence of membrane permeabilization. Figure 6 shows the release of β‐gal enzyme from *E. coli* after different contact times with CNFs. The results revealed that negative controls of untreated bacteria (black squares) showed no enzymatic activity, which was an indication of membrane integrity. When CNFs were added to the bacterial suspension, a progressive time‐dependent enzymatic activity was observed (red circles), a consequence of membrane lesion. However, results demonstrated that it was not possible to reach the maximum expected level of released β‐gal from chemically lysed cells (~ 420 β‐gal units). This suggests that the antibacterial effect of CNFs was not completed and the release of the enzyme is a longer process occurring after death and lysis of the cell. These results reasonably demonstrate the ability of CNFs to permeate bacterial membrane and coincide with the findings of Tao et al. (2011), who reported similar results for CS solutions.

### 3.6 Transmission electron microscopy analysis of membrane permeabilization effect of CNFs

**Figure 7:**
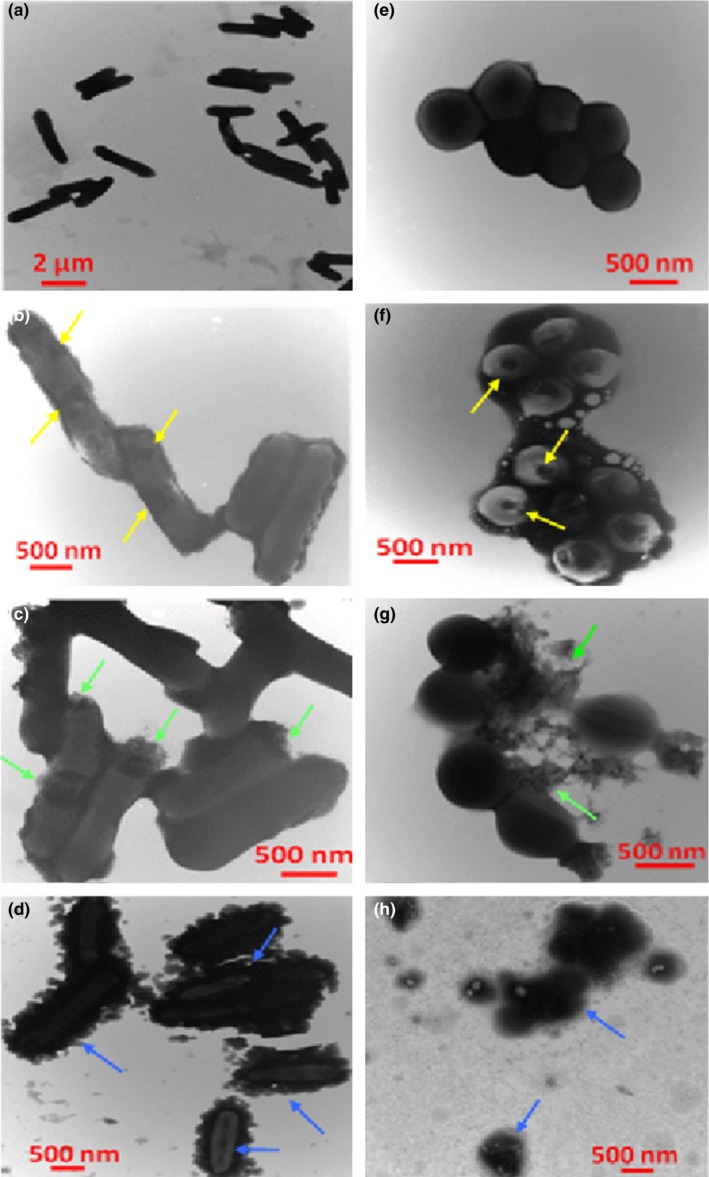
Transmission electron microscopy (TEM) micrographs of a, b, c, d: t_0_, 10, 20, and 30 min exposure of *E. coli* cells to CNFs, and e, f, g and h: t_0_, 10, 20, and 30 min exposure of *S. aureus* cells to CNFs, respectively. The yellow, green and blue arrows, respectively, point at membrane perforation, leakage of cytosol, and cell lysis

The effect of CNFs on membrane morphology and integrity was investigated by TEM (Figure 7). Untreated cells of *E. coli* (Gram‐negative) and *S. aureus* (Gram‐positive) were intact and did not show any membrane lesion or anomaly (Figure 7a and 6e). After exposure to CNFs, a remarkable alteration of membrane integrity was observed. TEM images of exposed cells to CNFs revealed that after 10 min contact, both *E. coli* and *S. aureus* strains showed membrane permeabilization by perforation (Figure 7b and f). After 20 min exposure, both bacteria were leaking cytosolic components (Figure 7c and g). However, membrane detachment occurred only in *E. coli* (Figure 7d). Gradual membrane detachment from the cell wall of *E. coli*, and shrinkage of the cytoplasm was observed after 30 min contact time with CNFs, as pointed by the blue arrows (Figure 7d). This detachment of the plasma membrane was due to desorption of the cytosol, subsequent to leakage of intracellular compounds (Figure 7c), making cells look transparent and empty (Figure 7d). After 30 min contact time, the cytoplasmic membrane of *E. coli* collapsed (Figure 7d) and *S. aureus* cells were completely disintegrated (Figure 7h). Adsorption of molecules to bacterial cell walls, of both *E. coli* and *S. aureus* was also observed and was proportional to contact time (Figure 7b, 7c and 7d). This might be due to (1) the release of intracellular components that can attach to the surface of bacteria, reflecting local cell rupture, or (2) to small soluble CS chains surrounding the bacterial cells *via* electrostatic and hydrophobic interactions, or (3) both possibilities. A simple visual inspection of the CS/PEO nanofiber mats, before and after the antibacterial tests indicated that the fibers were stable after 48 hr at 37°C, pH 5.8 in PBS. This suggests that resolubilization was only partial, as the mats remained intact. However, as nanofibers contain PEO, which is soluble in water, a certain solubility of PEO is expected. In addition, due to the pH of the medium (5.8), chitosan may solubilize partially, as verified by Ardila, daigle, Heuzey, and Ajji (2017). Consequently, both the released chitosan in the medium and the one remaining in the nanofiber mats may contribute to the antibacterial effect of the CNFs. The second conjuncture coincides with the findings of other authors (Chung et al., 2004; Helander, Nurmiaho‐Lassila, Ahvenainen, Rhoades, & Roller, 2001), who studied the adsorptive characteristics of bacterial cells to chitosan solutions. This suggests that the mechanism of action of CNFs may be also due to partial resolubilization of CS in the media, even though visually the mats looked intact after 48 hr in PBS or LB. Short CS chains might, thereby, penetrate the cell wall and perforate the plasma membrane, while longer chains could enclose bacteria and prevent cell exchange with the extracellular medium. Accordingly, Figure 7b, c, d, and f, show that CS formed an impermeable envelope surrounding the bacteria which might block the absorption of essential elements into the cells (Choi et al., 2001; Eaton, Fernandes, Pereira, Pintado, & Malcata, 2008). Ultimately, it can be inferred that the bactericidal effect of CNFs may be the result of membrane perforation. Our results are in agreement with those of other authors (Tao et al., 2011; Xing et al., 2009a), who observed membrane perforation of *E. coli* caused by CS solutions and particles, respectively. However, our experiments conducted on CNF‐treated *E. coli* (Gram‐negative) and *S. aureus* (Gram‐positive)*,* revealed various surface characteristics and cell stages in response to treatment with CNFs. This suggests that the mechanism of action of CNFs is a complex combination of different bactericidal effects that can occur at different stages: (1) CNFs inhibit bacterial growth through membrane pervasion and perforation, (2) partly resolubilized CS chains can kill bacteria by causing membrane rupture and/or suppressing cell exchange and nutrient uptake, (3) CS nanofibers and/or solutions can cause osmotic stress by chelating trace elements such as metallic ions, essential to bacterial growth. However, the common mechanism behind these different modes of action is undeniably due to the protonated functional groups of CS. The results clearly demonstrate that CNFs’ bactericidal effect involves permeabilization of bacterial membrane with pore formation, contrary to what has been reported so far. However, no evidence of penetration of the membrane can be inferred, even though pore formation assuredly occurred. The next challenge should aim at clarifying the molecular mechanisms behind the bactericidal activity of CNFs and identifying the membrane elements and metabolic pathways involved in the internalization of chitosan into the bacterial cell wall. These further studies will not only be critical for the application of such materials in food packaging, but also for the prevention of outbreak of resistance phenomena toward chitosan.

## 4 CONCLUSIONS

The results of this study show that the antibacterial activity of chitosan nanofibers (CNFs) can be attributed to membrane disruption and perforation. Consequently, this resulted in the leakage of intracellular components such as proteins and nucleotides. The bioavailability of NH_3_^+^ functional groups on CNFs favored and maximized cell adhesion and attachment to the surface of the mats. The model established here, regarding CNFs’ mode of action suggests that bacteria migrate to the surface of the nanofibers and not the reverse. Since bacteria use adhesion and attachment surfaces to better grow and multiply, CNFs showed the ability to efficiently attract and trap bacteria through electrostatic interactions, on account of their large surface‐to‐mass ratio and high porosity. Our results also suggest that adsorption of CS to the bacterial surface is the first step in CNFs’ mechanism of action, followed by membrane perforation, leakage of cytosolic compounds, and finally cell lysis and disintegration. Nevertheless, it is not excluded that part of the antibacterial activity might be due to partial dissolution of the nanofibers, making chitosan available in solution. As promising practical application, CNFs can be used as part of active food packaging in order to extend the shelf life of food products along with preventing spoilage by bacteria such as *E. coli*, and foodborne diseases caused by *Listeria*,* Staphylococcus* and *Salmonella*.

## CONFLICT OF INTEREST

None declared.
